# Prediction of preterm birth with and without preeclampsia using mid-pregnancy immune and growth-related molecular factors and maternal characteristics

**DOI:** 10.1038/s41372-018-0112-0

**Published:** 2018-05-24

**Authors:** Laura L. Jelliffe-Pawlowski, Larry Rand, Bruce Bedell, Rebecca J. Baer, Scott P. Oltman, Mary E. Norton, Gary M. Shaw, David K. Stevenson, Jeffrey C. Murray, Kelli K. Ryckman

**Affiliations:** 1 Department of Epidemiology and Biostatistics, University of California San Francisco School of Medicine, San Francisco, CA 94107 USA; 2 Department of Obstetrics, Gynecology and Reproductive Sciences, University of California San Francisco School of Medicine, San Francisco, CA 94107 USA; 3 Department of Pediatrics, University of Iowa School of Medicine, Iowa City, IA 52242 USA; 4 Department of Pediatrics, University of California San Diego, La Jolla, CA 92093 USA; 5 Department of Pediatrics, Stanford University School of Medicine, Stanford, CA 94305 USA; 6 Department of Epidemiology, University of Iowa, College of Public Health, Iowa City, IA 52242 USA; 7 California Preterm Birth Initiative, University of California San Francisco School of Medicine, San Francisco, California 94107 USA

## Abstract

**Objective:**

To evaluate if mid-pregnancy immune and growth-related molecular factors predict preterm birth (PTB) with and without (±) preeclampsia.

**Study design:**

Included were 400 women with singleton deliveries in California in 2009–2010 (200 PTB and 200 term) divided into training and testing samples at a 2:1 ratio. Sixty-three markers were tested in 15–20 serum samples using multiplex technology. Linear discriminate analysis was used to create a discriminate function. Model performance was assessed using area under the receiver operating characteristic curve (AUC).

**Results:**

Twenty-five serum biomarkers along with maternal age <34 years and poverty status identified >80% of women with PTB ± preeclampsia with best performance in women with preterm preeclampsia (AUC = 0.889, 95% confidence interval (0.822–0.959) training; 0.883 (0.804–0.963) testing).

**Conclusion:**

Together with maternal age and poverty status, mid-pregnancy immune and growth factors reliably identified most women who went on to have a PTB ± preeclampsia.

## Introduction

Worldwide, more than 15 million babies are born preterm (before 37 completed weeks of gestation) each year [1]. Preterm birth (PTB) and its related complications are the leading cause of death in children less than five years of age and contribute to more than one million deaths per year [2]. Survivors of PTB are more likely to suffer from both short- and long-term morbidities including blindness, deafness, neurodevelopmental delay, psychiatric disturbance, diabetes, and heart disease in later life [3–6]. While all neonates born preterm are at risk for short and long-term morbidity and mortality, those with early PTB (gestational age (GA), <32 weeks) are at the highest risk [3–8].

Despite increased clinical, research, and policy focus, rates of PTB are increasing worldwide—including in the United States [1]. After several years of decline, in 2015, the rate of PTB in the United States increased [9]. This pattern of increase continued into 2016 [10].

The continuing burden of PTB despite increased focus suggests the need for a different approach to addressing PTB from a research, clinical, and policy perspective. While historically, prevention efforts have focused mostly on women with a previous PTB or short cervix, or have focused on extending gestational duration in women with early signs of labor [11–13], there is a growing push for management based on a woman’s specific personal risk profile. In 2016, the Society for Maternal Fetal Medicine (SMFM) released its first PTB Toolkit [14] which outlines recommended management of women based on a number of risk factors for PTB (e.g., bacteriuria, smoking, obesity, pregestational diabetes, and chronic hypertension).

Consideration of a clinical shift to address the risk of PTB has also recently begun to be considered for women testing as “high-risk” based on mid-pregnancy biomarkers [15–17]. In general, the principle behind such tests is that they might allow for the identification of at-risk pregnant women that may otherwise go unidentified. While the question of whether women with molecular risk without other traditional risks (e.g., previous PTB, short cervix) might benefit from existing therapies (e.g., progesterone, cervical pessary, cervical cerclage, tocolytic administration, and antibiotic therapy) is still unknown, all of these interventions require timely administration [18]. These efforts are closely aligned with those focused on early identification of pregnancies at increased risk for preeclampsia (ending in preterm and term birth) given the established efficacy of aspirin administration ≤16-weeks for reducing recurrence [19, 20].

Recent years have seen progress in the development of PTB prediction tests with three tests in or moving into the market. Two existing tests measure proteins and microparticles identified by multiple reaction monitoring mass spectrometry [15, 16] and one uses circulating cell-free plasma RNAs tested by Q-PCR [17] to identify women at increased risk for spontaneous PTB. Currently these tests focus mostly on spontaneous PTB (PTB related to preterm premature rupture of membranes (PPROM) or premature labor) and generally do not address provider initiated PTB (PTB resulting from cesarean section or induction due to fetal or maternal indication). Efforts focused on molecular and other prediction testing for preeeclampsia are also well underway but also rarely address overlap with efforts aimed at predicting PTB [21, 22].

While existing prediction tests for spontaneous PTB (and for preeclampsia without a focus on PTB) demonstrate the promise of using mid-pregnancy biomarkers for prediction purposes, these tests lack a generalizability to all PTBs. Given the breadth of data demonstrating common pathophysiological underpinning across spontaneous and provider initiated subtypes of PTB including among those ± preeclampsia [23–28], it appears possible that a predictive test could be developed that covers a wider range of PTB phenotoypes. For example, all PTB subtypes ± preeclampsia have been shown to have strong links to markers of immune function (e.g., cytokines and chemokines) [23–26] and to angiogenic growth factors (e.g., vascular endothelial growth factor (VEGF)) [27, 28].

For this study, we hypothesized that a comprehensive test for PTB across subtypes including ± preeclampsia could be developed using mid-pregnancy growth and immune-related factors along with maternal demographics and obstetric factors. Markers were tested in 15–20 week serum samples collected as part of routine prenatal screening with predictive performance assessed in training and testing subsets.

## Materials and methods

All women included in the study are part of a population-based cohort of all singleton California births from July 2009 through December 2010 (*n* = 757,853). All women had gestational dating by first trimester ultrasound and had a second trimester serum marker test done as part of routine prenatal screening for aneuploidies and neural tube defects by the California Genetic Disease Screening Program (*n* = 241,000). Candidate cases and controls all had a second trimester serum sample banked by the California Biobank Program (*n* = 77,604) [29] and had detailed demographic and obstetric information available in a linked hospital discharge birth cohort database maintained by the California Office of Statewide Health Planning and Development (OSHPD) (*n* = 61,339). A number of previous papers have been published that leverage data and screening results for women in this and other California cohorts [30, 31]. The final source set for this study included 4025 singletons with births before 37 weeks, and 56,081 with births on or after 37 completed weeks through 44 weeks. From this set, we selected 100 PTB cases with gestational ages at birth <32 weeks, 100 PTB cases with gestational ages at birth from 32 to 36 weeks, and 200 term controls with gestational ages at birth from 39 to 42 weeks using simple random sampling wherein each within group pregnancy had an equal probability of selection. The resulting sample (by <32, 32–26, and 39 to 42 weeks) were then divided into training and testing subsets at a ratio of 2:1 (Supplemental Fig. 1). This was a convenient random sample wherein total number was determined based on the financial resources available for testing.

### Maternal demographic and obstetric characteristics

Demographic and obstetric factors evaluated included race/ethnicity, maternal age, years of formal education, place of maternal birth, low-income status (as indicated by “Medi-Cal” payment for delivery (the California health program for low-income persons (generally defined as income <138% of the United States poverty level)), parity, preexisting diabetes, preexisting hypertension, reported smoking, obesity (body mass index (BMI) ≥30 m/kg^2^), interpregnancy interval (IPI) <12 months, and previous PTB. All variables were derived from the OSHPD birth cohort file, which combines birth certificate records and all hospital discharge records for the mother and baby from 1 year prior to the birth to 1 year after the birth. Coding of preexisting and gestational diabetes and hypertension was based on International Classification of Diseases, 9th Revision, Clinical Modification (ICD-9-CM) [32] four digit codes contained in the cohort file.

### Serum biomarker testing

**Fig. 1:**
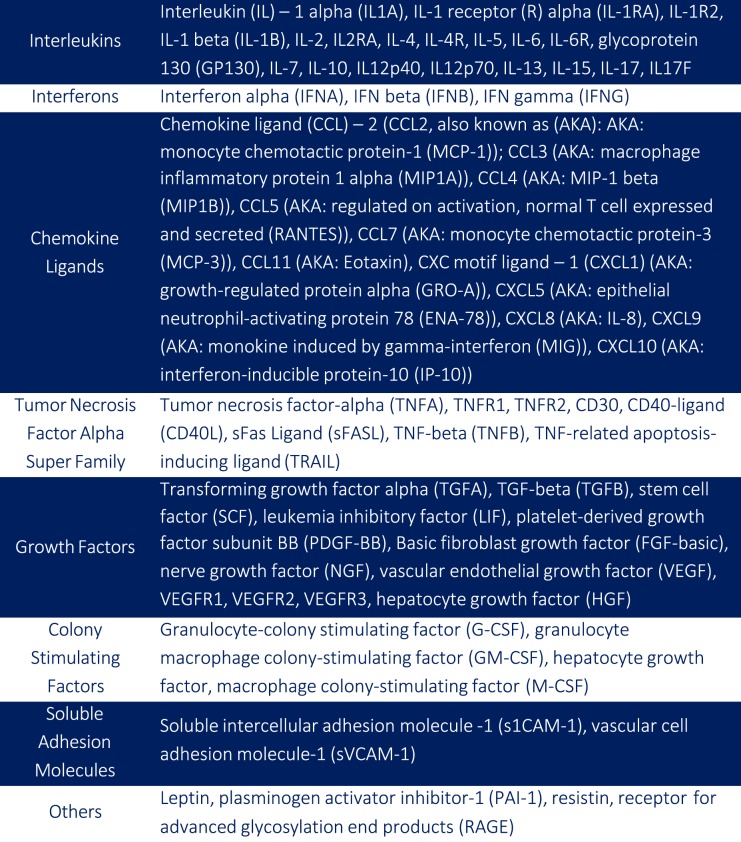
Serum markers measured in banked 15–20-week serum samples

Immune and growth-factor molecular testing was done using residual serum samples from second trimester (15–20 week) prenatal screening. Specimens were stored in 1 milliliter tubes at −80 °C. Markers tested included twenty interleukins, three interferons, eleven chemokine ligands, eight members of the tumor necrosis factor-alpha (TNFA) super family cytokines, 12 growth factors, three colony-stimulating factors, two soluble adhesion molecules, and leptin, plasminogen activator inhibitor-1 (PAI-1), resistin, and receptor for advanced glycosylation end products (RAGE) (see Fig. 1 for complete listing). While many of these markers have been shown to have close links to PTB or preeclampsia [24, 33–36], for this study we elected to run the full panel of immune and growth-factor related markers available via multiplex testing through our partner laboratory (the Human Immune Monitoring Center (HIMC) at Stanford University) [37] given the established interconnectedness of all of these markers to immune function and as such, the potential for revealing novel patterns and relationships—particularly given the role of immune function in pregnancy [38].

All markers were read using a Luminex 200 instrument (Austin, TX) in accordance with the manufacturer recommendations. Details regarding Luminex lab protocols at the HIMC are available on their website [37]. All markers were tested using a human multiplex kit that was purchased from Affymetrix Inc. (Santa Clara, CA) with the exception of human soluble receptors, which were measured using a Millipore high sensitivity multiplex kit (HSCRMAG32KPX14) (Billerica, MA). Median fluorescence intensity (MFI) values were reported for all markers using Masterplex software (Hitashi Solutions, San Bruno, CA). To avoid error inherent in log transformation of MFI to pg/mL, analyses relied on the MFI average, which was based on measurement of two aliquots tested on the same plate for each case and control. All inter-assay coefficients (CVs) were <15 % across all markers and all intra-assay CVs were <10%.

### Data analyses

Simple logistic regression (including odds ratios (ORs) and their 95% (CIs)) were used for association testing in the training set using demographic, clinical, and molecular factors (standardized using natural log transformation) and to build multivariate models. So as not to lose information that might be critical to prediction, for variable selection into multivariate models we utilized backward stepwise regression wherein all possible predictors were entered into the model and the criteria for remaining in the model was *p* < 0.20. Predictors with a *p* ≥ 0.05 and <0.20 were removed in any instance where their exclusion resulted in a <1% decrease in the concordance statistic (c-statistic) (equivalent to the area under the receiver operating characteristic curve (AUC)). Similarly, in any instance where the variable inflation factor (VIF) indicated major multicollinearity among predictors (defined as VIF ≥2.5) predictors were removed when their exclusion resulted in a <1% decrease in the c-statistic. All variables in the final multivariate logistic model were included in the final linear discriminate analysis (LDA) algorithm with assessment of performance using AUC in both the training and testing subsets. AUC performance was evaluated for all PTBs and for early PTB (<32 weeks) and late PTB (33–36) subgroups including in spontaneous and provider initiated subgroups and by preeclampsia diagnosis by ICD-9-CM code [32]. “Spontaneous PTBs” were considered to be those where the birth certificate or hospital discharge record noted ‘‘preterm premature rupture of membranes’’ (PPROM) or ‘‘preterm labor.’’ Pregnancies with a record of receiving tocolytics with no record of PPROM were also included in the preterm labor group. Pregnancies classified as “provider initiated” PTB were those without PPROM or premature labor for whom there was ‘‘medical induction’’, ‘‘assisted rupture of membranes’’, or for whom there was a cesarean delivery at <37 weeks of gestation and none of the aforementioned indicators of spontaneous PTB. Rates of PTB (overall and by subtypes and by preeclampsia) were examined by AUC derived probability scores (by deciles) to assess true- and false-positive performance at set cut-points in the training and testing subgroups.

All analyses were done using Statistical Analysis Software (SAS) version 9.3 (Cary, NC). Methods and protocols for the study were approved by the Committee for the Protection of Human Subjects within the Health and Human Services Agency of the State of California, the Institutional Review Board of Stanford University and the Institutional Review Board of the University of California San Francisco.

## Results

Most case and control women in the study identified themselves as Hispanic or White (e.g., 55.8% of women with a PTB delivery and 42.5% of women with a term delivery in the training sample were Hispanic and 47.5% of women with a PTB delivery and 42.5% of women with a term delivery in the testing sample were Hispanic). Most women in both the training and testing samples were between 18 and 34 years of age (67.5–75.0% across groupings). The majority women with a preterm delivery had a spontaneous PTB (82.5% in the training sample and 75.0% in the testing sample). The rate of preterm preeclampsia was 15.8% in the training sample and 22.5% in the testing sample (Table 1). Crude logistic analyses in the training sample revealed that women with PTB ± preeclampsia were significantly more likely (*p* < .05) than term controls to be low-income (as indicated by MediCal status) (OR 2.07, 95% CI 1.23–3.48) and to have lower MIP1B levels (OR 0.59, 95% CI 0.38–0.93) (Supplemental Table 1).

**Table 1:** Sample characteristics

	Training		Testing	
	PTB*n* (%)	Term*n* (%)	PTB*n* (%)	Term*n* (%)
Sample	120 (100.0)	120 (100.0)	80 (100.0)	80 (100.0)
Race/ethnicity				
Hispanic	67 (55.8)	51 (42.5)	38 (47.5)	34 (42.5)
White	39 (32.5)	49 (40.8)	26 (32.5)	35 (43.8)
Asian	8 (6.7)	9 (7.5)	11 (13.8)	5 (6.3)
Black	3 (2.5)	3 (2.5)	3 (3.8)	1 (1.3)
Other	0	1 (0.8)	2 (2.5)	0
Age (Years)				
<18	1 (0.8)	2 (1.7)	1 (1.3)	0
18–34	81 (67.5)	90 (75.0)	56 (70.0)	59 (73.8)
≥35	38 (31.7)	28 (23.3)	23 (28.8)	21 (26.3)
Other (all yes vs. no)				
<12 years education	22 (18.3)	21 (17.5)	16 (20.0)	11 (13.8)
Born in the United States	76 (63.3)	85 (70.8)	50 (62.5)	54 (67.5)
Low-Income^a^	61 (50.8)	40 (33.3)	35 (43.8)	30 (37.5)
Nulliparous	54 (45.0)	64 (53.3)	40 (50.0)	39 (48.8)
Reported smoking	3 (2.5)	2 (1.7)	1 (1.3)	1 (1.3)
Obese	29 (24.2)	21 (17.5)	18 (22.5)	10 (12.5)
Preexisting diabetes	3 (2.5)	1 (0.8)	4 (5.0)	1 (1.3)
Preexisting hypertension	7 (5.8)	3 (2.5)	10 (12.5)	0
Anemia	8 (6.7)	12 (10.0)	11 (13.8)	2 (2.5)
IPI < 12 Months	24 (20.0)	28 (23.3)	13 (16.3)	14 (17.5)
Preterm birth subgroups				
Spontaneous	99 (82.5)		60 (75.0)	
Provider initiated	17 (14.2)		18 (22.5)	
Subtype unknown	4 (3.3)		2 (2.5)	
<32 Weeks	60 (50.0)		40 (50.0)	
Spontaneous	53 (44.2)		32 (40.0)	
Provider initiated	5 (4.2)		8 (10.0)	
Subtype unknown	2 (1.7)		2 (2.5)	
32–36 Weeks	60 (50.0)		40 (50.0)	
Spontaneous	46 (38.3)		28 (35.0)	
Provider initiated	12 (10.0)		10 (12.5)	
Subtype unknown	2 (1.7)		2 (2.5)	
Preeclampsia (any)	19 (15.8)	2 (1.7)	18 (22.5)	1 (1.3)
<32 Weeks	9 (7.5)		13 (16.3)	
32–36 Weeks	10 (8.3)		5 (6.3)	

*IPI* interpregnancy interval

^a^Receiving assistance for medical services through the California MediCal program (requires an income of < 138% of federal poverty level)

The final 15 to 20 week PTB ± preeclampsia model included maternal age greater than 34-years and low-income status along with 25 serum biomarkers (Supplemental Table 2). Serum markers included eight interleukins (IL-1 receptor 2 (IL-1R2), IL-4, IL-4R, IL-5, IL-13, IL-17, IL-17F, and glycoprotein 130 (GP130)), one interferon (interferon (IFN) beta (IFNB)), one factor from the TNFA super family (sFAS ligand (sFASL)), five chemokine ligands (epithelial neutrophil-activating protein 78 (ENA-78), eotaxin, monokine induced by gamma-interferon (MIG), macrophage inflammatory protein 1 beta (MIP1B), and regulated on activation, normal T-cell expressed and secreted (RANTES)), five growth factors (stem cell factor (SCF), platelet-derived growth factor subunit BB (PDGF-BB), basic fibroblast growth factor (FGF-basic), nerve growth factor (NGF), and vascular endothelial growth factor R3 (VEGFR3)), two colony-stimulating factors (granulocyte-colony-stimulating factor (G-CSF), and macrophage colony-stimulating factor (M-CSF)), as well as PAI1, resistin, and RAGE. Although we found that many of the markers in the final model were highly correlated (VIFs ≥2.5 for 21 of the 24 markers in the final model (IL-1R2, IL-4, IL-5, IL-13, IL-17, IL-17F, GP130, IFNB, sFASL, ENA-78, eotaxin, MIG, MIP1B, SCF, PDGF-BB, FGF-basic, NGF, VEGFR3, G-CSF, M-CSF, and PAI1) (Supplemental Fig. 3), all of these markers contributed 1% or more to the c-statistic when included in the model and were, therefore, retained.

When considered in combination using the linear discriminate for PTB ± preeclampsia, the 25 target immune and growth factors along with maternal age >34 years and low-income status were able to identify more than 80% of women going on to deliver preterm in the training set (AUC 0.803, 95% CI 0.748–0.858) and 75.0% of women going on to deliver preterm in the testing set (AUC 0.750, 95% CI 0.676–0.825) (Table 2, Supplemental Fig. 2). Performance based on the use of combined maternal characteristics and serum markers exceed that based on the use of only characteristics or serum markers (AUC for all preterm birth using maternal age >34 and low-income status = 0.620, 95% CI (0.553–0.687) in the training set and AUC = 0.539 (95% CI 0.455–0.624) in the testing set; AUC for immune and growth markers only = 0.777 (0.719–0.835) in the training set and AUC = 0.743 (0.667–0.818) in the testing set. While performance varied some across PTB subgroups in the training and testing subsets, most AUCs were at or above 80%. One exception was in the training sample where the AUC for PTB 32–36 weeks was 0.790 (95% CI 0.718–0.862). The largest AUC observed was for preterm preeclampsia <32 weeks in the training sample (AUC = 0.953, 95% CI 0.728–0.881 with an AUC of 0.879 (95% CI 0.782–0.976 in the testing sample) (Table 2).

**Table 2:** Performance of mid-pregnancy immune and growth factor preterm birth ± preeclampsia test (overall and by preterm and preeclampsia subgroups)

	Training(*n* = 240)		Testing(*n* = 160)	
	AUC	95% CI	AUC	95% CI
All PTB	0.803	0.748–0.858	0.750	0.676–0.825
Spontaneous	0.806	0.748–0.864	0.837	0.770–0.903
Provider initiated	0.919	0.862–0.976	0.858	0.771–0.944
<32	0.837	0.777–0.897	0.806	0.717–0.896
Spontaneous	0.840	0.775–0.904	0.868	0.789–0.948
Provider initiated	0.927	0.818–1.000	0.878	0.738–1.000
34–36	0.790	0.718–0.862	0.827	0.748–0.906
Spontaneous	0.801	0.723–0.890	0.907	0.843–0.971
Provider initiated	0.932	0.871–0.995	0.893	0.796–0.989
Preeclampsia <37 weeks	0.889	0.822–0.959	0.883	0.804–0.963
<32 Weeks	0.953	0.728–0.881	0.879	0.782–0.976
32–36 Weeks	0.938	0.877–0.998	0.950	0.882–1.000

*sPTB* spontaneous preterm birth, *PPROM* preterm premature rupture of membranes, *AUC* area under the receiver operating characteristic curve

**Fig. 2:**
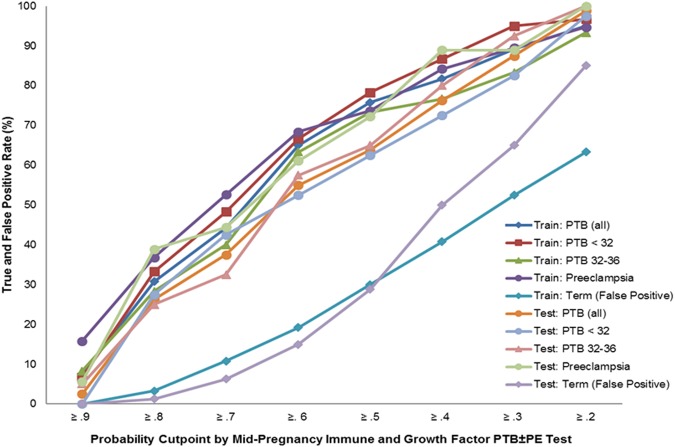
True and false-positive rates by probability cut-points based on mid-pregnancy immune and growth factor preterm birth ± preeclampsia test

LDA-derived probabilities from the PTB ± preeclampsia model yielded findings showing that the relationship between risk scores and PTB ± preeclampsia overall and by subtype was consistent across the training and testing subsets with improvements in detection at each lowering of the probability cut point also associated with an increase in term false positives (Fig. 2, Supplemental Table 3). Detection was generally better for PTBs <32 weeks and for preterm preeclampsia at each cut point than it was for PTBs from 32 to 36 weeks. For example, 30.8% of women with PTBs in the training sample and 27.5% of women with PTBs in the testing sample had probability scores ≥0.8 vs. 3.3% of women with term birth in the training sample and 1.3% of term birth in the testing sample (Fig. 2, Supplemental Table 3). Detection at this same cut point was best in women with a PTB <32 weeks and in women with preterm preeclampsia in both samples (33.3% in the training and 27.5% in the testing samples for PTB <32 weeks and 36.8% in the training sample and 38.9% in testing sample for preterm preeclampsia) (Fig. 2, Supplemental Table 3).

## Discussion

Results from this study show that when considered in combination, maternal characteristics and serum immune and growth-related markers can be used at 15–20 weeks of gestation to identify women at increased risk for PTB occurring ± preeclampsia. The resulting LDA PTB ± preeclampsia model was able to consistently identify more than three and four women going on to deliver preterm across training and testing subsets with the best performance for preterm preeclampsia where AUCs were consistently at or above 88%. LDA-derived probabilities were able to reliably specify a woman’s magnitude of risk for PTB ± preeclampsia with higher probabilities associated with lower term false-positive rates. For example, while >60% of women going on to have a PTB ± preeclampsia had a 15–20 week LDA-derived probability score ≥0.5 so did >28% of pregnancies going on to have a term delivery. While the detection rate was far lower at higher probability cut-points, so was the rate of false positives in term pregnancies. For instance at a LDA-derived probability score ≥0.8, detection rates for PTB were consistently above 25% and detection rates for PTB with preeclampsia were consistently above 35% with false-positive rates in pregnancies going to term that were consistently below 5%.

This is the first study that we are aware of that aimed to predict PTB across subtypes ± preeclampsia. Given that the AUCs from the present study equaled or exceeded those of investigations focused on, for example, spontaneous PTB [14–16] or preeclampsia [22] it appears that such an approach may offer similar predictive capacity and broader applicability over other serum testing approaches. For example, using circulating proteins, Saade and colleagues were able to identify women with a spontaneous PTB <37 weeks with an observed AUC of 0.75 [15] and Weiner and colleagues were able to identify women with a spontaneous PTB <37 weeks with an observed AUC of 0.76 using cell-free plasma RNAs [17] (compared with an AUC of 0.81 (rounded) for spontaneous PTB in the training set and 0.84 (rounded) in the testing set in the present study). Our results with respect to prediction of preterm preeclampsia also appear to meet or exceed other serum tests for preterm preeclampsia. For example, O’Gorman and colleagues reported an AUC of 0.95 for preeclampsia before 32 weeks and an AUC of 0.87 for any preeclampsia before 37 weeks using 11 to 13 week placental growth factor (PLGF) and pregnancy-associated plasma protein A (PAPP-A) [22]. We observed an AUC for preterm preeclampsia of 0.95 (rounded) in the training set and 0.88 (rounded) in the testing set for preeclampsia <32 weeks and we observed an AUC for all preterm preeclampsia (<37 weeks) of 0.89 in the training sample and 0.88 in the testing sample.

While the present test appears to perform as well or better for all births <37 weeks than other serum tests that are specific to spontaneous PTB and preeclampsia, two caveats to this should be noted. First, there is some evidence that -omics-based tests for spontaneous PTBs perform better at lower gestational ages than they do at higher gestational ages. Although based on smaller sample sizes for earlier PTBs than in the present study, Saade and colleagues [15] reported an AUC of 0.98 for <35 vs. ≥35 weeks and Catonwine and colleagues [16] reported an AUC of 0.89 for birth at ≤34 weeks. Also, prediction of preterm preeclampsia has been shown to be greatly improved if serum testing is combined with ultrasound measures of mean arterial pressure and uterine artery pulsatility index (AUCs 0.99 for preeclampsia <32 weeks and 0.92 for any preeclampsia <37 weeks) [22]. Although we contend that the currently presented algorithm represents an improvement over these other methods given that it focus on the commonalities across PTB subtypes and relies on widely available multiplex technology that allows multiple markers to be measured in a single test, it is critical to note that there are likely some benefits to focusing within subtypes. It may be that the present test could be improved further by the inclusion of, for example, a second-tier -omics-based test that addresses other protein-based or metabolic factors. A second-tier test that included ultrasound measures might also increase detection rates for preterm preeclampsia. Such an approach might allow for broad testing for baseline all PTB ± preeclampsia risk and second-tier testing that is specifically aimed at early PTBs and preterm preeclampsia with a focus on term false-positive reduction.

The present study focused on the capacity for prediction of PTB ± preeclampsia and as such, interpretation of causal underpinnings suggested by biomarker patterns should be approached with some caution. Still, given that the serum markers in the final model have established links with poor pregnancy outcomes and close ties to immune function and growth [23, 24, 27, 28, 33–36, 38–42] some insight into pathophysiological underpinnings is evident. Most notably, the findings from the current study are supportive of the role of perturbation of the cytokine network in the pathogenesis of PTB as proposed by Romero and colleagues [24]—particularly given that the success of the present model in prediction was driven by its reliance on a constellation of markers that were often highly related yet contributed independently to prediction. Study data also realized the assertions of others who have hypothesized that combining cross-pathway markers would increase test performance [43]. By combining cross-way molecular markers with risks like maternal age >34 years and low-income status, the model took advantage of well-established maternal risks for PTB [40] along with critical pathway signals.

Our findings of a persistent role in prediction for low-income status (including when defined by participation in state-sponsored health insurance programs for individuals with incomes near or below the United States poverty line) are consistent with other investigations [44, 45]. We suspect that in our model, poverty is serving as a proxy for unmeasured or underreported factors with links to PTB ± preeclampsia including, possibly, the presence of nutritional deficits, psycho-social or systemic stress, and greater exposure to potentially harmful substances like tobacco, alcohol, and pollution [44–46]. While we had information about tobacco and alcohol use (as well as drug use) in the study dataset, it is possible that these factors were underreported and as such, that the poverty flag is serving as a proxy for these factors as well as others that may be more common in lower income populations. These patterns support the need for additional research and clinical investigation with these women—particularly with respect to early and potentially modifiable risks. Whatever the case, It is important to note that in the present study these factors alone were poor predictors of preterm birth (with AUCs below 62% in the training and testing sets) and also that they contributed a relatively small amount of information over and above biomarkers alone (increasing the AUC for biomarkers only by 0.026+/−0.058 in the training set and by 0.008+/−0.075 in the testing set). As such, it is clear that these factors alone were not the sole drivers of overall risk and may point to more upstream drivers. Nevertheless, it is important to investigate these patterns more completely given potential for modification. These data also suggest that the efficacy of this test would not be diminished in settings characterized by mostly high- or low-income individuals given that molecular factors appear to be the primary drivers of prediction.

While the present study represents an improvement over other tests for PTB ± preeclampsia—particularly given applicability across PTB subgroups and to larger populations given the use of a random sampling design and the leveraging of multiplex technology available globally, there are important limitations of our work. Most notably, the present study relied on a fairly small number of cases and controls (*n* = 400). While the case number used in the present study is larger than those used to create other tests [21–23] it is still far too small to make firm conclusions about broad testing efficacy and performance. Also, because the current study relied on a population-based sampling design that reflected the true distribution of women participating in prenatal screening by race/ethnicity grouping, we were not powered to look at performance by all race/ethnicity groupings. Further replication and clinical validation are critical next steps especially in women of Black race/ethnicity and in women living in low-to-middle income countries given their higher risk for PTB [1, 9, 10, 44]. It is also important to note that because this study relied on women who had already elected to participate in prenatal screening for aneuploidies and neural tube defects, this sample is biased towards women who made that choice and as such, it is unclear is performance would be the same in all pregnant women.

Given that the model performed well with samples collected at as early as 15-weeks of gestation, we feel confident that the model could be applied at earlier gestational ages. Demonstrating such capacity will be critical given, for example, that the efficacy of aspirin administration to prevent preeclampsia has been shown to work the best if started at ≤16-weeks of gestation [19]. It will also be important to examine how this test behaves in identifying pregnancies that deliver early term (37 and 38 weeks). Given mounting data demonstrating that early term babies are at increased risk for both short- and long-term morbidity [47] and that these women are more likely to deliver preterm in the next pregnancy [48] it would be advantageous to be able to identify these women early in pregnancy in an effort to extend gestation.

It is also important to address some statistical and model complexity issues raised by the current analyses. Specifically, it is notable that the present model is more complex than those that rely on fewer factors. While we do not believe this issue is of considerable consequence given that all markers can be tested simultaneously using multiplex technology (and as such, this complexity has minimal impact on how much serum would be needed for the test or how much it would cost), it does raise some issues with respect to model transparency and comprehension. We have presented the full LDA function used for classification to address this issue (Table 3), however, the fact does remain that the algorithm we developed would have to be applied to any woman’s biomarker testing results before they could be used clinically—likely via some electronic platform. It is also of note that some markers in the final model—namely FGF-basic and IL-4 exhibited a particularly large influence on the PTB ± preeclampsia algorithm while also having large observed confidence intervals in initial multivariate logistic models (Supplemental Table 2). Both of these factors were normally distributed after log transformation and as such, the large risks and confidence intervals observed appeared to be driven by the separation of values for these markers in cases vs. controls after adjustment for the other factors in the model. Given this and the contribution of both to AUC performance these factors were kept in the model, however, these patterns are of note and should be kept in mind as replication and clinical validation efforts move forward. It is possible that in other samples such separation may not be evident and as such, some cost to the performance of the model could occur. No such cost appeared to transpire in the testing set but nonetheless, behavior of these markers should be tracked as efforts move forward. In addition, it should be recognized that because many of the markers in the model are highly correlated but were retained due to their individual contribution to the c-statistic, future larger studies may find that not all markers are required for maximum test performance. To this point, it should also be recognized that any interpretation of underlying etiology based on individual marker-specific findings should be avoided given that the model presented is focused on prediction and is based on a markers and maternal factors considered in combination.

**Table 3:** Final 15–20 week linear discriminate for preterm birth (PTB) ± preeclampsia^a^

	No preterm birth/PE	Preterm birth/PE
Constant	−2229	−2207
PAI1	413.49597	411.87715
Resistin	0.75258	1.88708
GP130	119.61108	118.44810
ENA-78	−29.26997	−28.53583
sFASL	5.54682	4.15190
FGF-basic	200.03457	204.35713
G-CSF	10.37429	10.68791
IL-1R2	−2.50083	−2.23721
IL-4	−97.38072	−94.75076
IL-4R	23.32864	22.69110
IL-5	65.86996	63.28213
IL-13	−35.04245	−33.45918
IL-17	−114.44812	−113.34045
IL-17F	−1.80384	−2.20769
IFNB	4.26576	3.87186
M-CSF	−46.88392	−47.52238
NGF	8.44649	6.96815
PDGFBB	−23.52635	−22.59093
RAGE	−4.15909	−3.75774
SCF	40.47520	37.72616
VEGFR3	14.01668	13.74962
Eotaxin	−51.73581	−53.79304
MIG	5.47441	5.91727
MIP1B	16.13980	14.87844
RANTES	5.15387	4.74134
Age > 34 years	−15.30541	−14.42951
Low-income^b^	3.66412	4.71827

^a^Results presented to the fifth decimal point to allow for complete transparency and replication of complete algorithm

^b^Receiving assistance for medical services through the California MediCal program (requires an income of <138% of the federal poverty level)

Along with maternal age and poverty status, mid-pregnancy immune and growth factors measured by a single test reliably identified women who went on to have a PTB ± preeclampsia. Such information has the potential to be used to identify women who may benefit from existing and emerging interventions aimed at reducing rates of PTB and preeclampsia [49].

## Electronic supplementary material

Supplemental Table 1

Supplemental Table 2

Supplemental Table 3

Supplemental Figure 1

Supplemental Figure 2

Supplemental Figure 3
