# Correction: Prediction of preterm birth with and without preeclampsia using mid-pregnancy immune and growth-related molecular factors and maternal characteristics

**DOI:** 10.1038/s41372-018-0158-z

**Published:** 2018-06-25

**Authors:** Laura L. Jelliffe-Pawlowski, Larry Rand, Bruce Bedell, Rebecca J. Baer, Scott P. Oltman, Mary E. Norton, Gary M. Shaw, David K. Stevenson, Jeffrey C. Murray, Kelli K. Ryckman

**Affiliations:** 1 Department of Epidemiology and Biostatistics, University of California San Francisco School of Medicine, 94107 San Francisco, CA USA; 2 Department of Obstetrics, Gynecology and Reproductive Sciences, University of California San Francisco School of Medicine, 94107 San Francisco, CA USA; 3 Department of Pediatrics, University of Iowa School of Medicine, 52242 Iowa City, IA USA; 4 Department of Pediatrics, University of California San Diego, 92093 La Jolla, CA USA; 5 Department of Pediatrics, Stanford University School of Medicine, 94305 Stanford, CA USA; 6 Department of Epidemiology, University of Iowa, College of Public Health, 52242 Iowa City, IA USA; 7 California Preterm Birth Initiative, University of California San Francisco School of Medicine, 94107 San Francisco, CA USA

Correction to: *Journal of Perinatology*; 10.1038/s41372-018-0112-0; published online 24 May 2018

This Article was originally published under Nature Research's License to Publish, but has nowbeen made available under a [CC BY 4.0] license. The PDF and HTML versions of the Articlehave been modified accordingly.

