# Supplementary material for: Prediction of preterm birth with and without preeclampsia using mid-pregnancy immune and growth-related molecular factors and maternal characteristics
Source: J Perinatol. 2018 May 24;38(8):963–72. doi: 10.1038/s41372-018-0112-0 (PMC6089890; doi:10.1038/s41372-018-0112-0)
Supplement: Supplementary file 1 — Supplemental Table 1 [file 41372_2018_112_MOESM1_ESM.docx]

Supplemental Table 1. Crude odds ratios, training set: Demographic, clinical, and serum biomarkers in term births versus preterm births ± preeclampsia (all serum markers log transformed).

|  | Odds Ratio | 95% CI | p = |
| --- | --- | --- | --- |
| Race/ethnicity^a^ |  |  |  |
| Hispanic | 1.65 | 0.95 – 2.88 | 0.08 |
| Asian | 1.12 | 0.39 – 3.16 | 0.83 |
| Black | 1.26 | 0.24 – 6.57 | 0.79 |
| Age (Years)^b^ |  |  |  |
| < 18 | 0.56 | 0.05 – 6.24 | 0.63 |
| ≥ 35 | 1.50 | 0.85 – 2.66 | 0.16 |
| Other^c^ |  |  |  |
| < 12 Years Education | 1.06 | 0.55 – 2.05 | 0.87 |
| Born in the United States | 0.71 | 0.41 – 1.22 | 0.22 |
| Low Income^d^ | 2.07 | 1.23 – 3.48 | <0.01 |
| Nulliparous | 0.72 | 0.43 – 1.19 | 0.20 |
| Reported Smoking | 1.51 | 0.25 – 9.22 | 0.65 |
| Obese | 1.50 | 0.80 – 2.82 | 0.21 |
| Preexisting Diabetes | 3.05 | 0.31 – 29.76 | 0.34 |
| Preexisting Hypertension | 2.42 | 0.61 – 9.57 | 0.21 |
| Anemia | 0.64 | 0.25 – 1.63 | 0.35 |
| IPI < 12 Months | 0.82 | 0.44 – 1.52 | 0.53 |
| Interleukins^e^ |  |  |  |
| IL-1A | 0.95 | 0.70 – 1.27 | 0.71 |
| IL-1RA | 0.88 | 0.58 – 1.33 | 0.53 |
| IL-1R2 | 1.02 | 0.82 – 1.27 | 0.87 |
| IL-1B | 1.00 | 0.88 – 1.13 | 0.99 |
| IL-2 | 1.00 | 0.78 – 1.29 | 0.99 |
| IL-2RA | 0.89 | 0.58 – 1.37 | 0.59 |
| IL-4 | 1.02 | 0.85 – 1.23 | 0.82 |
| IL-4R | 0.82 | 0.55 – 1.21 | 0.31 |
| Il-5 | 0.67 | 0.37 – 1.20 | 0.18 |
| IL-6 | 0.83 | 0.58 – 1.17 | 0.28 |
| IL6R | 0.72 | 0.38 – 1.38 | 0.32 |
| GP130 | 0.94 | 0.79 – 1.12 | 0.46 |
| IL-7 | 0.77 | 0.45 – 1.32 | 0.34 |
| IL-10 | 0.99 | 0.79 – 1.25 | 0.96 |
| IL-12p40 | 0.96 | 0.80 – 1.14 | 0.62 |
| IL-12p70 | 0.72 | 0.39 – 1.34 | 0.30 |
| IL-13 | 0.95 | 0.69 – 1.31 | 0.77 |

(Continued)

Supplementary Table 1 (continued).

|  | Odds Ratio | 95% CI | p = |
| --- | --- | --- | --- |
| Interleukins^e^ |  |  |  |
| IL-15 | 0.98 | 0.72 – 1.33 | 0.88 |
| IL-17 | 0.99 | 0.75 – 1.31 | 0.95 |
| IL17F | 1.00 | 0.90 – 1.11 | 0.99 |
| Interferons^e^ |  |  |  |
| IFNA | 0.99 | 0.87 – 1.12 | 0.83 |
| IFNB | 0.99 | 0.87 – 1.13 | 0.88 |
| IFNG | 1.01 | 0.90 – 1.13 | 0.88 |
| Chemokine Ligands^e^ |  |  |  |
| MCP1 | 1.02 | 0.87 – 1.20 | 0.78 |
| MIP1A | 0.90 | 0.77 – 1.05 | 0.17 |
| MIP1B | 0.59 | 0.38 – 0.93 | 0.02 |
| RANTES | 0.91 | 0.71 – 1.18 | 0.47 |
| MCP3 | 0.98 | 0.79 – 1.22 | 0.86 |
| Eotaxin | 1.01 | 0.82 – 1.24 | 0.93 |
| GRO-A | 1.01 | 0.88 – 1.15 | 0.90 |
| ENA-78 | 1.00 | 0.71 – 1.41 | 0.98 |
| IL-8 | 1.02 | 0.90 – 1.16 | 0.73 |
| MIG | 1.06 | 0.90 – 1.25 | 0.52 |
| IP-10 | 1.01 | 0.72 – 1.41 | 0.95 |
| Tumor Necrosis Factor Alpha Super Family^e^ |  |  |  |
| TNFA | 0.97 | 0.79 – 1.21 | 0.80 |
| TNFR1 | 0.70 | 0.40 – 1.21 | 0.20 |
| TNFR2 | 0.86 | 0.40 – 1.84 | 0.69 |
| CD30 | 1.01 | 0.73 – 1.40 | 0.95 |
| CD40L | 0.82 | 0.62 – 1.08 | 0.16 |
| sFASL | 0.96 | 0.79 – 1.18 | 0.72 |
| TNFB | 0.99 | 0.85 – 1.15 | 0.85 |
| TRAIL | 0.87 | 0.60 – 1.28 | 0.49 |
| Growth Factors^e^ |  |  |  |
| TGFA | 0.97 | 0.79 – 1.21 | 0.80 |
| TGFB | 1.03 | 0.84 – 1.26 | 0.79 |
| SCF | 0.97 | 0.75 – 1.25 | 0.82 |
| LIF | 1.02 | 0.85 – 1.23 | 0.82 |
| PDGFBB | 0.87 | 0.63 – 1.20 | 0.39 |
| FGF-Basic | 1.01 | 0.71 – 1.44 | 0.97 |
| NGF | 0.47 | 0.21 – 1.05 | 0.07 |
| VEGF | 0.95 | 0.66 – 1.35 | 0.76 |
| VEGFR1 | 0.97 | 0.88 – 1.08 | 0.61 |
| VEGFR2 | 0.95 | 0.82 – 1.09 | 0.45 |
| VEGFR3 | 0.96 | 0.83 – 1.12 | 0.63 |
| HGF | 1.00 | 0.79 – 1.27 | 0.99 |

(continued)

Supplementary Table 1 (continued).

|  | Odds Ratio | 95% CI | p = |
| --- | --- | --- | --- |
| Colony Stimulating Factors^e^ |  |  |  |
| G-CSF | 1.06 | 0.89 – 1.27 | 0.57 |
| GM-CSF | 0.93 | 0.67 – 1.30 | 0.67 |
| M-CSF | 0.97 | 0.79 – 1.19 | 0.77 |
| Soluble Adhesion Molecules^e^ |  |  |  |
| sICAM1 | 0.90 | 0.70 – 1.15 | 0.38 |
| sVCAM1 | 1.15 | 0.86 – 1.54 | 0.35 |
| Others |  |  |  |
| Leptin | 0.84 | 0.63 – 1.12 | 0.23 |
| PAI1 | 1.02 | 0.75 – 1.38 | 0.91 |
| Resistin | 1.11 | 0.73 – 1.70 | 0.63 |
| RAGE | 1.16 | 0.82 – 1.65 | 0.41 |
| CI, Confidence interval  ^a^  Odds ratio computed with White race/ethnicity as referent.  ^b^ Odds ratio computed with 18-34 years of age as referent.  ^c^  Odds ratio computed as yes versus no.  ^d^ Receiving assistance for medical services through the California MediCal program (requires an income of < 138% of the federal poverty level).  ^e^ See Figure 1 for full biomarker names. | | | |
