# Supplementary material for: Prediction of preterm birth with and without preeclampsia using mid-pregnancy immune and growth-related molecular factors and maternal characteristics
Source: J Perinatol. 2018 May 24;38(8):963–72. doi: 10.1038/s41372-018-0112-0 (PMC6089890; doi:10.1038/s41372-018-0112-0)
Supplement: Supplementary file 2 — Supplemental Table 2 [file 41372_2018_112_MOESM2_ESM.docx]

Supplemental Table 2. Markers from multivariate logistic model included in final linear discriminate for preterm birth ± preeclampsia.

|  | Odds Ratio | 95% CI | p = |
| --- | --- | --- | --- |
| PAI1 | 0.14 | 0.01 – 1.62 | 0.12^c^ |
| Resistin | 3.15 | 1.53 – 6.48 | 0.01 |
| GP130 | 0.29 | 0.10 – 0.82 | 0.02 |
| ENA-78 | 2.12 | 0.06 – 4.24 | 0.02 |
| sFASL | 0.21 | 0.06 – 0.70 | 0.01 |
| FGF-Basic | 66.74 | 3.02 – >999.99 | 0.01 |
| G-CSF | 1.48 | 0.81 – 2.70 | 0.19^c^ |
| IL-1R2 | 1.40 | 0.87 – 2.25 | 0.17^c^ |
| IL-4 | 15.68 | 1.04 – 236.90 | 0.04 |
| IL-4R | 0.55 | 0.27 – 1.12 | 0.10^c^ |
| IL-5 | 0.09 | 0.01 – 1.17 | 0.07^c^ |
| IL-13 | 6.57 | 0.91 – 47.67 | 0.06^c^ |
| IL-17 | 5.28 | 0.73 – 37.93 | 0.10^c^ |
| IL-17F | 0.67 | 0.38 – 1.19 | 0.17^c^ |
| IFNB | 0.67 | 0.36 – 1.23 | 0.19^c^ |
| M-CSF | 0.47 | 0.18 – 1.21 | 0.12^c^ |
| NGF | 0.08 | 0.01 – 0.96 | 0.05 |
| PDGFBB | 3.01 | 1.23 – 7.39 | 0.02 |
| RAGE | 1.63 | 0.94 – 2.82 | 0.08^c^ |
| SCF | 0.05 | 0.01 – 0.43 | 0.01 |
| VEGFR3 | 0.70 | 0.47 – 1.04 | 0.08^c^ |
| Eotaxin | 0.13 | 0.01 – 2.18 | 0.16^c^ |
| MIG | 1.64 | 1.00 – 2.68 | 0.05 |
| RANTES | 0.62 | 0.38 – 1.02 | 0.06^c^ |
| Age > 34 Years | 2.58 | 1.24 – 5.36 | 0.01 |
| Low Income^b^ | 2.80 | 1.44 – 5.45 | < 0.01 |

^a^ For logistic model there were no p-value limits on entry, retention at p < .20 with further exclusion where decrease in area under the Receiver Operating Characteristic curve (AUC) was < 1.0%.

^b^ Receiving assistance for medical services through the California MediCal program (requires an income of < 138% of federal poverty level).

^c^ Factor included in model despite p >.05 given that removal resulted in a ≥ 1.0 % decrease in AUC.
