# Supplementary material for: Prediction of preterm birth with and without preeclampsia using mid-pregnancy immune and growth-related molecular factors and maternal characteristics
Source: J Perinatol. 2018 May 24;38(8):963–72. doi: 10.1038/s41372-018-0112-0 (PMC6089890; doi:10.1038/s41372-018-0112-0)
Supplement: Supplementary file 3 — Supplemental Table 3 [file 41372_2018_112_MOESM3_ESM.docx]

Supplemental Table 3. Frequency of preterm birth ± preeclampsia overall and by timing subgroup and term birth by probability cut points generated by the linear discriminate function.

|  | Training Sample (n = 240) | | | | | Testing Sample (n = 160) | | | | |
| --- | --- | --- | --- | --- | --- | --- | --- | --- | --- | --- |
|  | Preterm | | | | Term | Preterm | | | | Term |
|  | < 37w | < 32w | 32-36w | PE |  | < 37w | < 32w | 32-36w | PE |  |
|  | n =  (%) | n =  (%) | n =  (%) | n =  (%) | n =  (%) | n =  (%) | n =  (%) | n =  (%) | n =  (%) | n =  (%) |
|  |  |  |  |  |  |  |  |  |  |  |
| Sample | 120  (100.0) | 60  (100.0) | 60  (100.0) | 19  (100.0) | 120  (100.0) | 120  (100.0) | 60  (100.0) | 60  (100.0) | 18  (100.0) | 120  (100.0) |
| ≥ .9 | 9  (7.5) | 4  (6.7) | 5  (8.3) | 3  (15.8) | 0 | 2  (2.5) | 0 | 2  (5.0) | 1  (5.6) | 0 |
| ≥ .8 | 37  (30.8) | 20  (33.3) | 17  (28.3) | 7  (36.8) | 4  (3.3) | 21  (26.3) | 11  (27.5) | 10  (25.0) | 7  (38.9) | 1  (1.3) |
| ≥ .7 | 53  (44.2) | 29  (48.3) | 24  (40.0) | 10  (52.6) | 13  (10.8) | 30  (37.5) | 17  (42.5) | 13  (32.5) | 8  (44.4) | 5  (6.3) |
| ≥ .6 | 78  (65.0) | 40  (66.7) | 38  (63.3) | 13  (68.4) | 23  (19.2) | 44  (55.0) | 21  (52.5) | 23  (57.5) | 11  (61.1) | 12  (15.0) |
| ≥ .5 | 91  (75.8) | 47  (78.3) | 44  (73.3) | 14  (73.7) | 36  (30.0) | 51  (63.8) | 25  (62.5) | 26  (65.0) | 13  (72.2) | 23  (28.8) |
| ≥ .4 | 98  (81.7) | 52  (86.7) | 46  (76.7) | 16  (84.2) | 49  (40.8) | 61  (76.3) | 29  (72.5) | 32  (80.0) | 16  (88.9) | 40  (50.0) |
| ≥ .3 | 107  (89.2) | 57  (95.0) | 50  (83.3) | 17  (89.5) | 63  (52.5) | 70  (87.5) | 33  (82.5) | 37  (92.5) | 16  (88.9) | 52  (65.0) |
| ≥ .2 | 114  (95.0) | 58  (96.7) | 56  (93.3) | 18  (94.7) | 76  (63.3) | 79  (98.8) | 39  (97.5) | 40  (100.0) | 18  (100.0) | 68 (85.0) |
| < .2 | 6  (5.0) | 2  (3.3) | 4  (6.7) | 1  (5.3) | 44  (36.7) | 1  (1.3) | 1  (2.5) | 0 | 0 | 12  (15.0) |

Abbreviations: w, weeks; PE, preeclampsia.
