# Supplementary figures and images for: Prediction of preterm birth with and without preeclampsia using mid-pregnancy immune and growth-related molecular factors and maternal characteristics
Source: J Perinatol. 2018 May 24;38(8):963–72. doi: 10.1038/s41372-018-0112-0 (PMC6089890; doi:10.1038/s41372-018-0112-0)

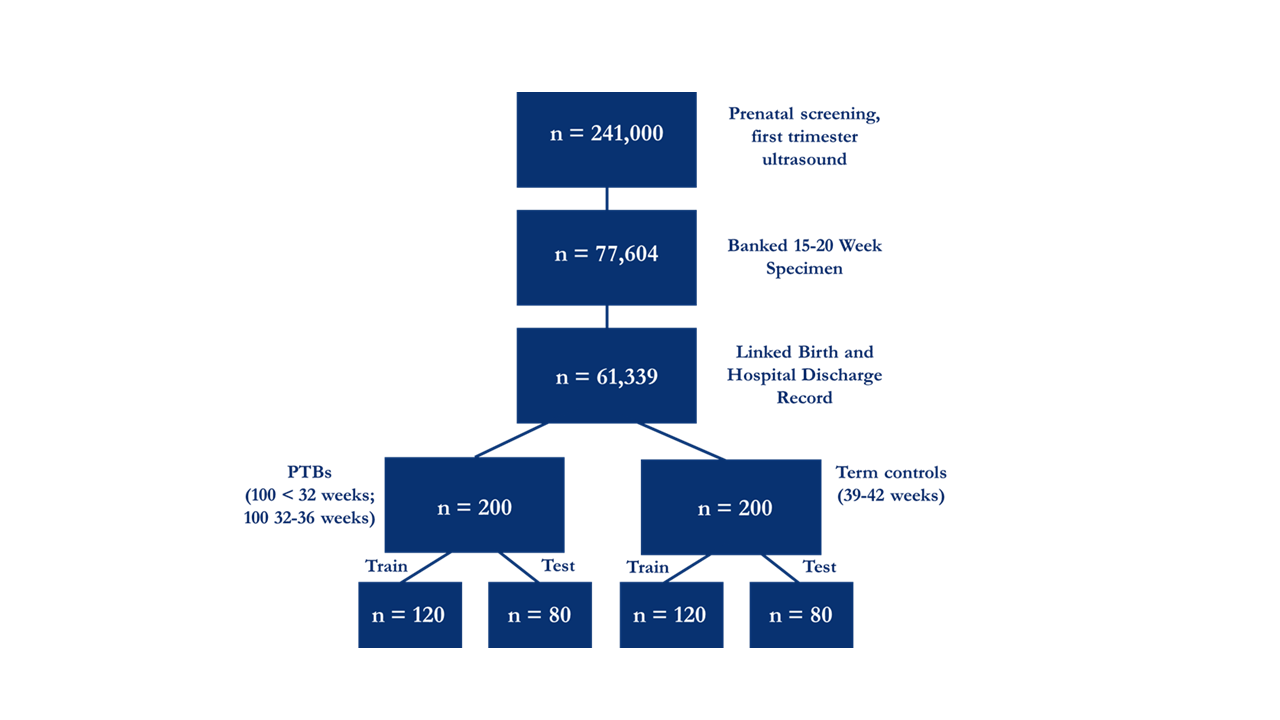

Supplement: Supplementary file 4 — Supplemental Figure 1 [file 41372_2018_112_MOESM4_ESM.tif]

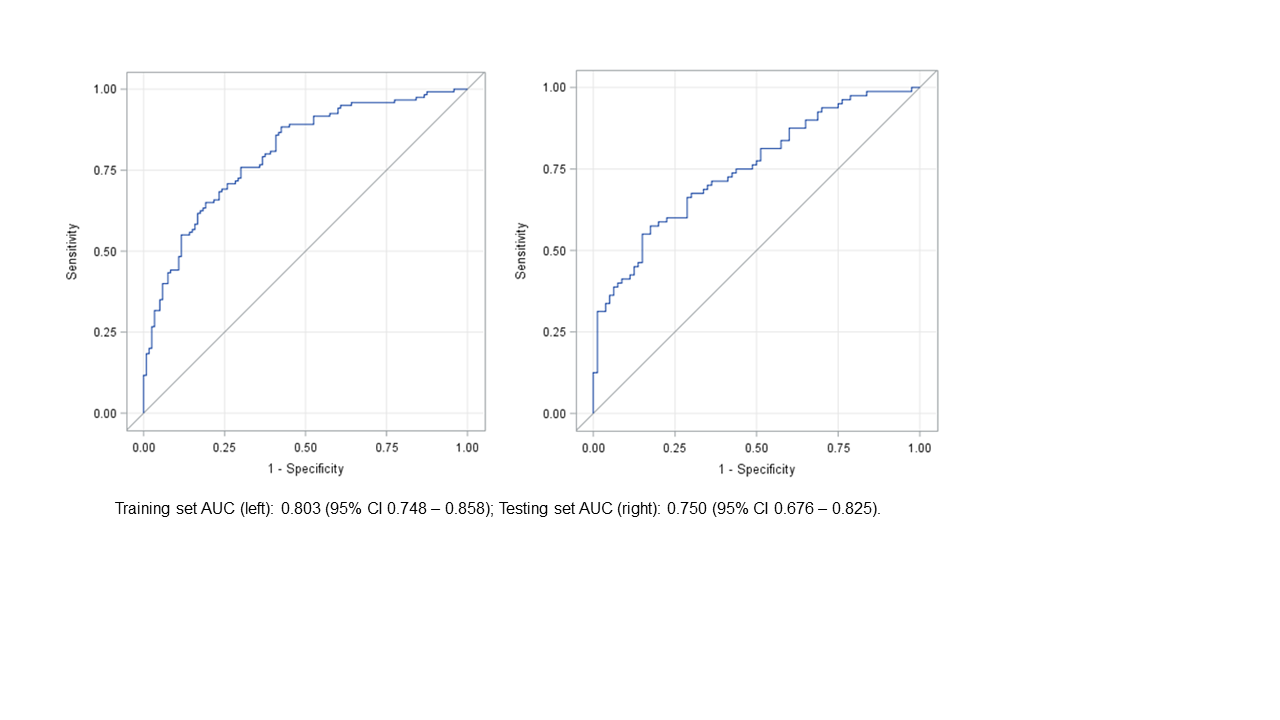

Supplement: Supplementary file 5 — Supplemental Figure 2 [file 41372_2018_112_MOESM5_ESM.tif]

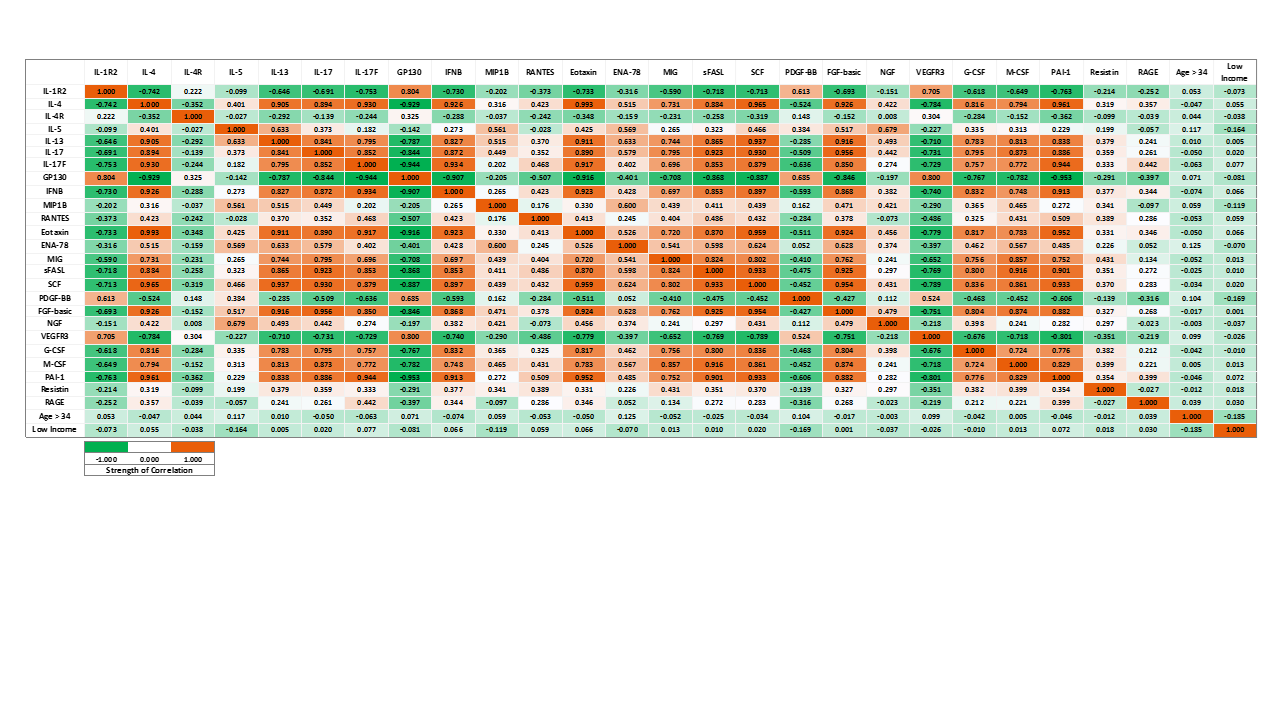

Supplement: Supplementary file 6 — Supplemental Figure 3 [file 41372_2018_112_MOESM6_ESM.tif]
